# Decreased Tertiary Lymphoid Structures in Lung Adenocarcinomas with ALK Rearrangements

**DOI:** 10.3390/jcm11195935

**Published:** 2022-10-08

**Authors:** Yi Zou, Jing Zhao, Fengbo Huang, Xueping Xiang, Yang Xia

**Affiliations:** 1 Department of Pathology, Second Affiliated Hospital of Zhejiang University School of Medicine, Hangzhou 310009, China; 2 Department of Medical Oncology, Second Affiliated Hospital of Zhejiang University School of Medicine, Hangzhou 310009, China; 3 Key Laboratory of Respiratory Disease of Zhejiang Province, Department of Respiratory and Critical Care Medicine, Second Affiliated Hospital of Zhejiang University School of Medicine, Hangzhou 310009, China; 4 Cancer Center, Zhejiang University, Hangzhou 310058, China

**Keywords:** lung neoplasms, anaplastic lymphoma kinase, tertiary lymphoid structures, tumor microenvironment, immunohistochemistry

## Abstract

Purpose: This study sought to characterize the tumor immune microenvironment (TIME) of lung adenocarcinomas with ALK rearrangements (ALK+ LUAD), which responds poorly to immune checkpoint inhibitors (ICIs) therapy. Materials and methods: Immune score evaluation and immunohistochemical (IHC) validation of B cells, cytotoxic, helper, regulatory T cells, dendritic cells, and tumor-associated macrophages were performed on the TCGA cohort and the whole tissue sections of our matched surgical samples, respectively, between ALK+ and ALK− LUAD. The formation and spatial organization of TLS, intra- and extra-TLS immune cell features, and tumor PD-L1 expression were analyzed independently. Results: Immune scores and TLS-signature gene levels were found to be lower in ALK+ TCGA LUAD. Quantitative IHC comparison confirmed the lower densities of TLS (0.10/mm^2^ vs. 0.34/mm^2^, *p* = 0.026) and intra-TLS immune cells (CD4+ helper T cells: 57.65/mm^2^ vs. 274.82/mm^2^, *p* = 0.026; CD8+ cytotoxic T cells: 22.46/mm^2^ vs. 172.83/mm^2^, *p* = 0.018; and CD20+ B cells: 36.08/mm^2^ vs. 207.29/mm^2^, *p* = 0.012) in ALK+ surgical samples. The TLS formation was negatively correlated with tumor progression in ALK+ tumors. The proportion of intra-TLS CD8+ cytotoxic T cells was the independent protective factors of node metastasis (HR: 0.599, 95% CI: 0.414–0.868, *p* = 0.007), and the density of intra-TLS CD20+ B cells was the independent protective factor of pStage (HR: 0.641, 95% CI: 0.446–0.922, *p* = 0.016). Tumors with intratumoral TLS showed significantly higher expression of PD-L1 (*p* = 0.029). Conclusion: ALK+ LUAD harbored a cold TIME featured by decreased TLS formation, which closely correlated to tumor progression and might contribute to the poor efficiency of ICIs.

## 1. Introduction

Non-small-cell lung cancer (NSCLC) is a highly malignant tumor with top mortality worldwide. The discovery of oncogenic driver genes and corresponding targeted drugs dramatically shifted the paradigm of lung cancer treatment. The rearrangement of the anaplastic lymphoma kinase (ALK) gene was a crucial driver genetic event, identified in 3–7% of NSCLC patients, and first-line ALK-tyrosine kinase inhibitors (TKIs) have markedly improved the patients’ survival benefit compared to conventional chemotherapy. Despite the excellent efficacy of ALK-TKIs, patients inevitably acquire resistance to ALK inhibitors due to ALK secondary resistance mutations or amplification, activation of bypass tracks, and lineage changes [1].

Immune checkpoint inhibitors (ICIs) are essential treatment options for NSCLC. However, data from previous RCTs and retrospective studies consistently revealed that the clinical benefit of single-agent ICIs is extremely poor in ALK+ NSCLC. In the phase II ATLANTIC study, no responses were observed in 15 ALK+ lung cancer patients treated with durvalumab as a back-line regimen. A real-world study also showed a 0% response rate in 23 ALK+ NSCLC patients [2]. Inline in the early-stage NSCLC scenario, such as IMPOWERE 010 study, the median disease-free survival (mDFS) was not improved by the adjuvant atezolizumab in the ALK+ subgroup but was numerically elevated in EGFR mutated subgroup [3]. Moreover, the mDFS of the atezolizumab group was even shorter than the patients receiving the best supportive care [3]. A few reports tried to decipher the poor responsiveness to PD-1/PD-L1 inhibitors in the ALK+ subgroup from the perspective of tumor immune microenvironment (TIME). They found low PD-L1 expression and low CD8+ T cell infiltration [4,5,6] in ALK+ NSCLC, potentially through the activation of PI3K, MAPK, and Hippo pathways [7,8], However, some inconsistent or even opposite results regarding PD-L1 expression and CD8+ T cell infiltration were also revealed by other studies [7,9,10]. Notably, the underline relationship between the TIME in ALK+ tumors and the poor response to ICIs would go beyond PD-L1 and CD8+ T cells. As important peripheral schools of lymphocytes, tertiary lymphoid structures (TLS), which are defined as ectopic lymphoid structures [11], have proved significant on the clinicopathological features or prognosis in many solid tumors, including NSCLC [12]; however, the characteristics of TLS and its significance in ALK+ tumors have not been studied before.

Hence, we dug the data from The Cancer Genome Atlas (TCGA) database in the current study first. Then, we comprehensively depicted the TIME of ALK+ tumors using our surgically resected lung adenocarcinoma (LUAD) specimens. TLS immune cells (TLS-IC) and extra-TLS immune cells (ETLS-IC), including tumor-infiltrating lymphocytes (TILs) and tumor associated macrophages, were evaluated independently. The crosstalk among these immune cells was also analyzed.

## 2. Materials and Methods

### 2.1. Data Availability

The gene expression, mutation, and clinical data of lung adenocarcinoma (LUAD) cases in The Cancer Genome Atlas (TCGA) were downloaded from cBioPortal (https://www.cbioportal.org/study/summary?id=luad_tcga_pan_can_atlas_2018, accessed on 9 August 2021). Gene rearrangements in LUAD cases were identified using Pipeline for RNA-Sequencing Data Analysis [13,14].

According to the alterations of known driver genes, cases with the mutation(s) of EGFR, ERBB2, MET, BRAF, KRAS, or any combinations of the genes above (MUL) were classified into non-rearrangement-mutated (NRM) group, those with the rearrangement of ALK, ROS1, NTRK, or RET were classifieds into gene-rearranged (GR) group. Those without any genetic alterations above were classified into the not-otherwise-specified (NOS) group. To balance the sample size of GR and non-GR (NRM+NOS) groups, propensity score matching (PSM) was performed before comparisons, according to age, sex, and pathological stages (pStage) using the matching ratio of 1:10 and the caliper width of 0.05.

The tumor immune microenvironment of each case was evaluated based on the signature gene expression of TLS [15,16], and each immune cell with the algorithm of XCELL [17] on TIMER 2.0 [18,19,20]. The comparison of gene expression and immune cells among groups was performed on the online platforms of SangerBox, and the cluster analyses were conducted on the online platform of Morpheus.

### 2.2. Patient Selection and Propensity Score Matching

Pathological reports of patients who underwent radical lung cancer resection in the Second Affiliated Hospital of Zhejiang University School of Medicine (SAHZJU) were reviewed. ALK+ cases between 2017 and 2018 were selected according to the following criteria: i. neoadjuvant chemotherapy and (or) immunotherapy had not been applied, ii. the pathological diagnosis was primary LUAD, and iii. the immunohistochemical (IHC) staining and fluorescence in situ hybridization (FISH) assay for ALK were all positive (details were seen in IHC staining and FISH assay).

According to the following criteria, ALK− cases were selected from LUAD with available next-generation sequencing (NGS) data in 2018: (i). neoadjuvant chemotherapy and (or) immunotherapy had not been applied; (ii). the pathological diagnosis was primary LUAD; (iii). the immunohistochemical (IHC) staining and fluorescence in situ hybridization (FISH) assay for ALK were all negative (details were seen in IHC staining and FISH assay); (iv). specific gene mutations were not found in NGS, including EGFR, ERBB2, MET, BRAF, KRAS, or any combinations of the genes above (MUL).

TIME comparison was performed between matched ALK+ and ALK− cases by PSM. To balance the patient baseline characterized by demographic and clinicopathological features, PSM was performed according to age, sex, and pStage using the matching ratio of 1:1 and the caliper width of 0.05. The significance of TIME features in ALK+ LUAD was evaluated in all ALK+ cases.

Patients’ demographic and clinicopathological data, including sex, age, tumor size, histologic differentiation, aerogenic spread, lymphovascular and pleural invasion, lymphatic and distant metastasis, were retrieved from the Electronic Pathological Workstation or Electronic Medical Record System of SAHZJU. The pTNM stage of each case was determined based on the American Joint Committee on Cancer (AJCC) cancer staging system (eighth).

This study was approved by the Review Board of Second Affiliated Hospital of Zhejiang University School of Medicine (2021–0562); patient consent was waived by the institutional review boards, as this study was retrospective, and patients’ information was protected by a blind method.

### 2.3. IHC Staining and FISH Assay

The ALK IHC assay was performed on the Ventana platform with the D5F3 antibody. Positive and negative controls were set for each case simultaneously. The IHC slides were reviewed and evaluated by two attending pathologists (Y. Z. and F. H.) according to VENTANA ALK (D5F3) CDx Assay Interpretation Guide for Non-Small Cell Lung Carcinoma. Briefly, only the cases showing strong and granular cytoplasmic staining in tumor cells were interpreted as positive.

In immunohistochemistry, B cells, dendritic cells (DC), tumor-associated macrophages (TAM), T helper (Th) cells, cytotoxic T lymphocytes (CTL), and regulatory T (Treg) cells were labeled by CD20, CD11c, CD163, CD4, CD8, and FOXP3, respectively. A tissue block containing the largest tumor section (when tumor size ≤ 3 cm) or containing tumor and adjacent normal tissue (when tumor size > 3 cm) was chosen as the representative block for each case. Eight 4 μm serial sections were cut and stained with antibodies against CD8 (SP16, ZSGB-Bio, Beijing, China, ready-to-use In Vitro Diagnostic kit), CD4 (EPR6855, Abcam, Boston, USA, 1:500), CD20 (L26, Maxim tech, Fuzhou, China, ready-to-use In Vitro Diagnostic kit), FOXP3 (EPR22102-37, Abcam, Boston, MA, USA, 1:250), CD45RO (UCH-L1, Abcam, Boston, MA, USA, 1:200), CD11c (EP1347Y, Abcam, Boston, MA, USA, 1:500), CD163 (EPR19518, Abcam, Boston, MA, USA, 1:500), and PD-L1 (28-8, Abcam, Boston, MA, USA, 1:500). In addition, an extra hematoxylin & eosin (H&E) slide of each representative section was also prepared.

Positive and negative cases for ALK in IHC were confirmed by the FISH assay. According to standard protocols, the FISH assays for ALK arrangement were performed on tissue sections with a Vysis ALK Break Apart FISH kit (Abbott Molecular, Abbott Park, IL, USA). Briefly, the LSI ALK 5′ probe (Green) and the LSI ALK 3′ probe (RED) were labeled, hybridized, and read along with standard controls. One was defined as a positive case when the proportion of positive cells (with separate green and red signals or individual red signals) were more than 50%, and at least 50 tumor cells should be evaluated for each case.

### 2.4. Quantitative Analysis

All slides were converted into digital images using Aperio Digital Pathology Slide Scanners (Aperio Technologies, Vista, CA, USA). The areas of tumors were measured with ImageJ (version 1.53k, National Institutes of Health, Bethesda, MD, USA).

For each case, TLS was defined as CD20+ cell aggregates larger than 60,000 μm [2,21], of which location, count, and area were evaluated based on CD20 staining with ImageJ (version 1.53k, National Institutes of Health, Bethesda, MD, USA) by one-by-one annotating. Their distribution was classified as intratumoral (within the invasive margin) or peritumoral (on or outside the invasive margin, but in direct contact with the tumor) type, based on the locations of most TLS. The number of main TLS immune cells, TLS-ICs (tB cells, tTh cells, and tCTL), were measured with ImageJ (version 1.53k, National Institutes of Health, Bethesda, MD, USA), based on which TLS density, single-TLS size, the ratio of areas between TLS and the tumor (TLS/tumor), the densities of main TLS-ICs in tumors, the proportions of main TLS-ICs in TLS (tB cell%, tTh cell%, and tCTL%) were calculated. In addition, the proportions of minor TLS-ICs (tTreg cell%, tDC%, and tTAM%) were graded semi-quantitatively with the cutoffs of <1%, 1–10%, and 10–50%, referring to the guidance of the International Immuno-Oncology Biomarkers Working Group [22].

In addition, extra-TLS CD4+ Th cells (eTh cells) and CD8 + CTL (eCTL) were designated as TILs [22], which constituted extra-TLS immune cells (ETLS-ICs) with extra-TLS TAM (eTAM). Five 200X fields were selected randomly in each case to measure the densities of eTh cells, eCTL, and eTAM with ImageJ (version 1.53k, National Institutes of Health, Bethesda, MD, USA). The PD-L1 expression of tumor cells (TC) was graded as TC < 1%, TC ≥ 1%, and TC ≥ 10% by two pathologists (F. H. and Y. Z.) double-blindly.

### 2.5. Statistical Analysis

The distribution of categorical variables, such as demographic, clinicopathological features, and ranked variables of TLS or stromal immune cells, were compared using the 𝜒^2^ test or Fisher’s exact test. Quantitative variables of TLSs were presented as means ± SEM and compared with the T-test if they followed normal or approximately normal distributions. Those following abnormal distributions were presented as medians (range) and compared with the U test. Correlation and risk analysis were performed with the Spearman test or binary logistic regression. The crosstalk chart was created with BioRender.com. A *p* value less than 0.05 was considered statistically significant. Statistical analyses and PSM were performed with SPSS 26.0 (SPSS Inc., Chicago, IL, USA).

## 3. Results

### 3.1. Lower Immune Scores and TLS-Signature-Gene Expression in TCGA ALK+ LUAD

**Figure 1:**
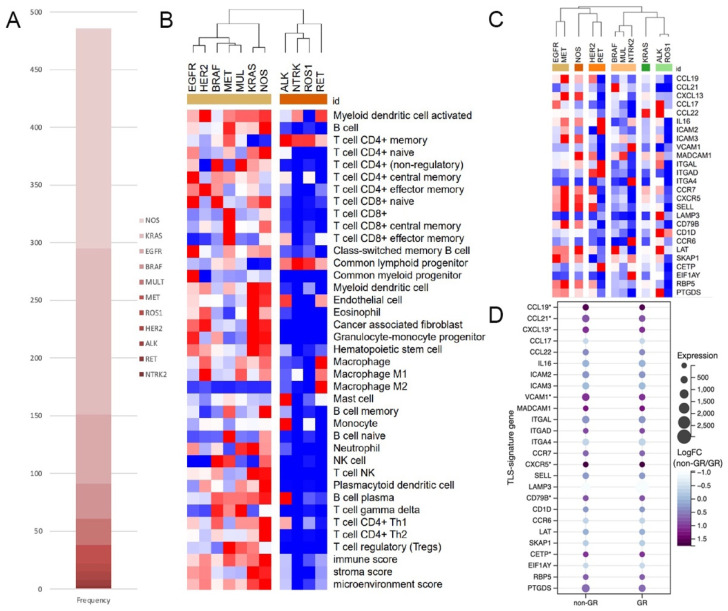
ALK+ LUAD had significantly lower immune scores and TLS-signature-gene expression. (**A**). Composition of genetic changes in TCGA cohort; (**B**). Heat map of immune scores of different mutants, (**C**,**D**). Heat map of the expression of TLS-signature genes (*: *p* < 0.05, NOS: not-otherwise-specified).

The RNA-seq data of 543 LUAD cases were obtained online, in which 486 cases had their mutational data available. Out of the patients, 295/486 (60.7%) harbored at least one of the genetic alterations concerned in this study, 15 cases had gene rearrangements, among which ROS1 (7/15) was the most common gene involved, followed by ALK (5/15), RET (2/15), and NTRK2 (1/15). An additional 280 cases with non-rearrangement mutations concerned here were classified into the non-rearrangement-mutated group. The most common genetic changes occurred in the KRAS gene (144/280), followed by the EGFR (60/280), BRAF (30/280), multiple (MUL) (23/280), MET (16/280), and HER2 gene (7/280). The remaining 191 cases with no specific driver-gene aberration were classified into the NOS group (Figure 1A).

In clustering analysis based on immune scores, all subgroups of gene rearrangements were categorized into the same cluster, dramatically differing from all subgroups in the non-rearrangement-mutated group (Figure 1B). But when clustered by the expression TLS-signature gene, subgroups in the gene-rearranged group could not get together well (Figure 1C). However, in the comparison of TLS-signature gene expression between matched GR and non-GR cases (Supplementary Figure S1 for details), all differentially expressed genes, including CCL19, CCL21, CXCL13, VCAM1, CXCR5, CD79B, and CETP, were downregulated in the GR group (Figure 1D).

### 3.2. LUAD Cohort from SAHZJU

A total of 39 patients who met the criteria were evaluated in this study. Patients’ age ranged from 27 to 80 years with a median of 58 years, and no sex predominance was found. Tumor sizes were distributed between 0.6–5.0 cm, and 22/39 (56.4%) tumors were moderately differentiated (Table 1).

There were eleven ALK− LUAD patients with available tissue blocks were adopted as candidate control cases, and PSM was used to balance the baseline between ALK+ and ALK− cohorts. Candidate control patients’ age ranged from 37 to 71 years with a median of 55 years, and no sex predominance was found. Out of these patients, six (66.0%), three (33.0%), and two (22.0%) were classified into pStage I, II, or III, respectively (Table 1). By PSM, differences of age, sex, and pStage between ALK-positive and -negative patients were ultimately diminished, and 18 matched patients were obtained for the following analyses (Supplementary Table S1).

### 3.3. Lower TLS Density and Fewer TLS-ICs in ALK+ LUAD

**Figure 2:**
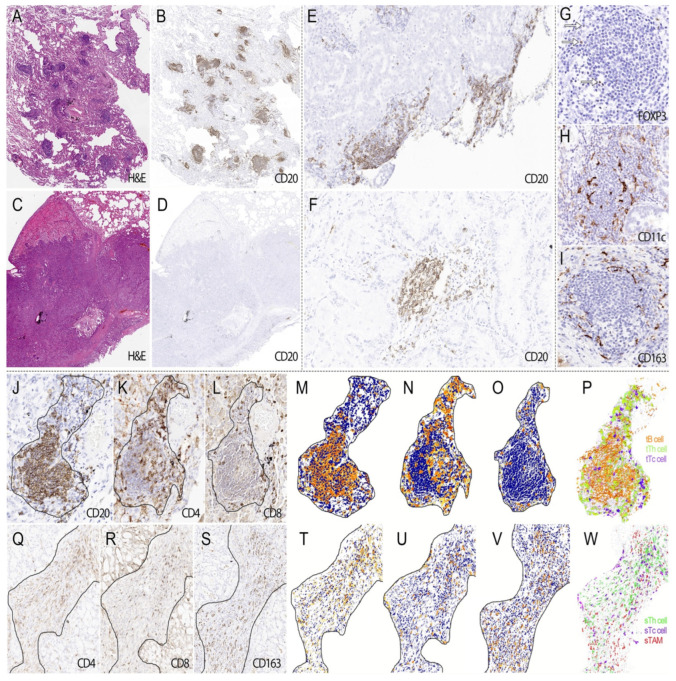
The morphological features of TLS and ETLS-ICs in surgical samples. TLS presents in tumors in different amounts ((**A**). H&E ×40; (**B**). CD20 ×40; (**C**). H&E ×40, and (**D**). CD20 ×40), distributing around or within tumors ((**E**). CD20 ×40 and (**F**). CD20 ×100). In IHC staining, TLS was mainly composed of B cells ((**J**). CD20 ×200) and Th cells (**K**). CD4 ×200), as well as some CTL ((**L**). CD8 ×200). Treg cells (**G**). arrow, FOXP3 ×400) were distributed in the peripheral region of TLS, but the latter was much sparser than the former. Both DC ((**H**). CD11c ×400) and TAM (**I**). CD163 ×400) had ramified cell bodies, and the former went deeper into TLS than the latter’s peripheral distribution. Images after processing and reconstruction make it more apparent that B cells (**M**,**P**) tended to arrange in nests of different sizes, with or without germinal centers, surrounded by Th cells (**N**,**P**) and CTL (**O**,**P**). In the stroma, TILs ((**Q**). CD4 ×200 and (**R**). CD8 ×200) and TAM ((**S**). CD163 ×200) account for the majority of ETLS-ICs. The corresponding processed and reconstructed image showed that Th cells (**T**,**W**) tended to distribute in the central region of the stroma, while CTL (**U**,**W**) were more closed to the tumor tissue. TAM (**V**,**W**) intertwined with the immune cells above without a specific distribution pattern.

In our surgical samples, TLS is present in 82% of patients. The TLS count per slide ranged from 1 to 58, with a median of 15. Most of them could be identified on H&E slides as aggregates of lymphocytes, which were located either around tumors or within tumors (Figure 2A–F), and their cellular components were highlighted by IHC staining (Figure 2G–P). In tumor stroma, TILs and eTAM accounted for most ETLS-ICs, and T cells rather than B cells predominantly constituted the TILs (Figure 2Q–W).

**Figure 3:**
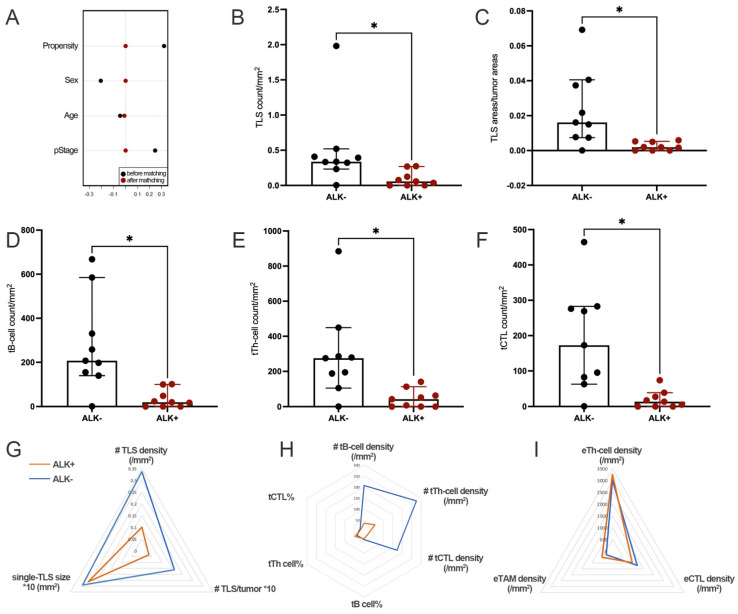
ALK+ LUAD had significantly lower TLS density and fewer TLS-ICs. (**A**). The propensity scores before and after PSM; (**B**). Comparison of the TLS count per tumor area; (**C**). Comparison of the ratio of areas between TLS and the tumor; (**D**). Comparison of tB-cell density; (**E**). Comparison of tTh-cell density; (**F**). Comparison of tCTL density; (**G**). Comparison of TLS basic features; (**H**). Comparison of TLS-ICs; (**I**). Comparison of ETLS-ICs. (*, #: *p* < 0.05).

Significant TIME differences were found between well matched ALK+ and ALK− tumors (Figure 3A). Compared to ALK− tumors, ALK+ tumors showed significantly weaker TLS formation, reflected by the lower TLS density (Figure 3B,G), lower TLS/tumor (Figure 3C,G), and lower densities of all main TLS-ICs, including tB cells (Figure 3D,H), tTh cells (Figure 3E,H), and tCTL (Figure 3F,H). However, there was no significant difference of the single-TLS size and the proportions of TLS-ICs between ALK+ and ALK− tumors (Figure 3G).

The densities of stromal immune cells, including eTh cells, eCTL, and eTAM, as well as tumor PD-L1 expression, were also comparable between tumors with or without ALK rearrangement (Figure 3I, Supplementary Table S2).

### 3.4. Significant Correlations between TIME Features and the Progression of ALK+ LUAD

**Figure 4:**
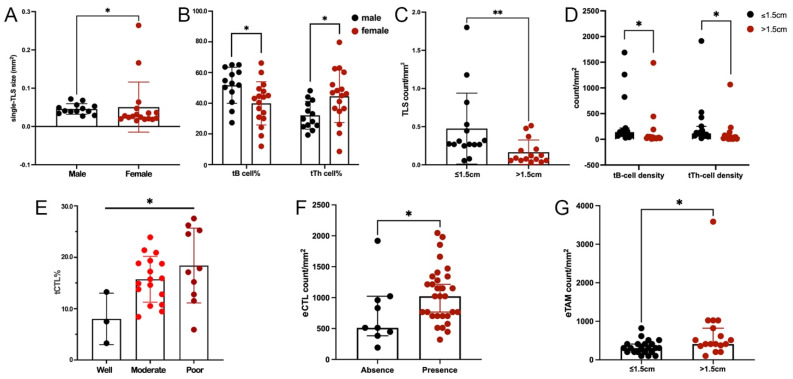
TIME features correlated to clinicopathological features of ALK+ LUAD. (**A**). Comparison of single-TLS size between sex; (**B**). Comparison of tB cell% and tTh cell% between sex; (**C**). Comparison of TLS density between tumors in different sizes; (**D**). Comparison of tB-cell and tTh-cell densities between tumors in different sizes; (**E**). Comparison of tCTL% between tumors with different differentiations; (**F**). Comparison of eCTL density between tumors with and without TLS; (**G**). Comparison of eTAM density between tumors in different sizes. (*: *p* < 0.05, **: *p* < 0.001).

TLS was found in 30 out of 39 tumors. Male patients had significantly larger single-TLS size (Figure 4A) and higher tB cell% (Figure 4B), but lower tTh cell% (Figure 4B). Large tumors (lager than the median size, >1.5 cm) had significantly lower densities of TLS (Figure 4C), tB cells, and tTh cells (Figure 4D). tCTL% was negatively associated with tumor differentiation (Figure 4E). However, the presence or location of TLS and tCTL density were comparable among different demographic or clinicopathological features (Supplementary Tables S3–S5), and there was no significant finding regarding the grades of tTreg cell%, tDC%, and tTAM% (Supplementary Tables S6–S8).

In extra-TLS regions, higher eCTL or eTAM density was found in tumors with TLS (Figure 4F) or in large sizes (Figure 4G). eTh-cell density was comparable among different demographic and clinicopathological features (Supplementary Table S9).

The significance of TLS and ETLS-IC features on tumor size, node metastasis, and pStage of ALK+ LUAD was examined by logistic regressions. Firstly, significant variables regarding TLS basic features, TLS-IC-related features, and ETLS-IC-related features were screened out from Model 1, 2, and 3, respectively, and then evaluated together in the final models. It was shown that TLS-related variables showed stronger powers than ETLS-ICs: a lower tCTL% was the independently significant risk factor for node metastasis, and a lower tB-cell density was the independently significant risk factor for higher pStage. However, no significant correlation was found between tumor size and TLS or ETLS-IC features (Table 2, Supplementary Tables S10–S12).

### 3.5. Close Interactions among TLS, ETLS-ICs, and PD-L1 in ALK+ LUAD

**Figure 5:**
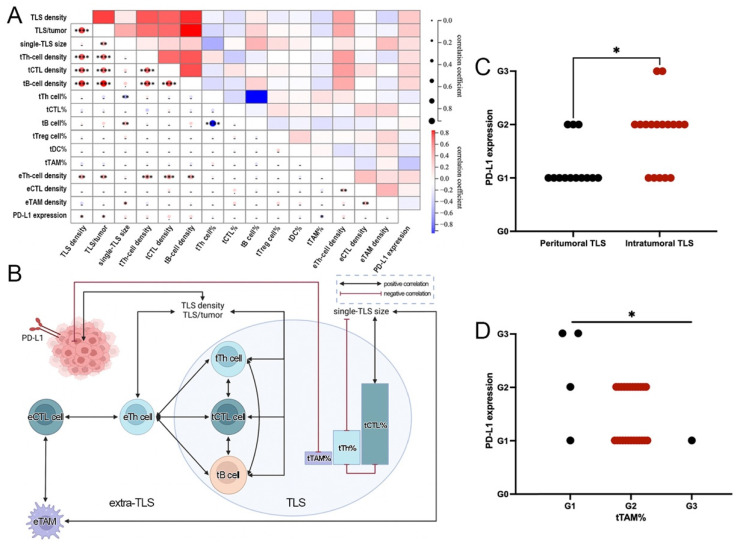
Close interactions among TLS, ETLS-ICs, and PD-L1 in ALK+ LUAD. (**A**). Heat map of the correlation between different immune parameters; (**B**). Crosstalk among different immune parameters; (**C**). Comparison of PD-L1 expression between tumors with different TLS distribution; (**D**). Comparison of PD-L1 expression between tumors with different tTAM grades. (* *p* < 0.05, ** *p* < 0.01, *** *p* < 0.001, **** *p* < 0.0001).

Significant correlations were found between TLS and ETLS-IC features: eTh-cell density was positively related with TLS density, TLS/tumor, or TLS-IC densities, and eTAM density was positively associated with single-TLS size (Figure 5A,B).

More correlations were found among TLS or ETLS-IC features. Among TLS features, TLS density was positively correlated with TLS/tumor and the densities of main TLS-ICs but was irrelevant to single-TLS size. However, single-TLS size was positively associated with tB cell% but negatively with tTh cell%, consistent with the negative correlation between tB cell% and tTh cell%. In addition, the densities of main TLS-ICs were positively relevant to each other (Figure 5A and B). Among ETLS-IC features, eTh-cell density had positive correlations with the densities of other major ETLS-ICs except for eTAM, but eTAM density showed a positive correlation with eCTL density (Figure 5A,B).

Of the ALK+ tumors, 49.23% (20/39) were positive for PD-L1 (TC ≥ 1%). In addition, 16 (41.0%) and 4 (10.3%) cases showed TC ≥1% and <10%, or TC ≥10%, respectively. No differential expression of PD-L1 was seen between tumors with and without TLS. However, more tumors with intratumoral TLS distributed in higher TC grades than those with peritumoral TLS (Figure 5C), and more tumors with lower tTAM% grades expressed higher levels of PD-L1 (Figure 5D). PD-L1 expression showed positive correlations with TLS density or TLS/tumor, and a negative correlation with tTAM% (Figure 5A,B). Apart from the above, there was no significant difference in PD-L1 expression among tumors with different TLS and ETLS-IC features (Supplementary Table S13).

Based on the above, crosstalk could be identified among TLS, ETLS-ICs, and PD-L1 in ALK+ LUAD, and eTh cells played as a bridge between TLS and ETLS-IC features (Figure 5A,B).

## 4. Discussion

As far as we know, this is the first systemic study focusing on TLS in ALK+ LUAD. ALK+ tumors harbored a relatively cold TIME, characterized by significantly lower expression of TLS-related genes, less TLS formation, and impaired TLS development. TLS-related factors, especially their cellular components, were independently protective factors against tumor progression, indicating the potential predominance of humoral immunity in ALK+ tumors, which might help explain the poor efficacy of blocking the PD-1/PD-L1 pathway.

Although ALK inhibitors have significantly improved the clinical outcome of patients with ALK-rearranged NSCLC, acquired resistance remains a significant challenge. Salvage chemotherapy, such as platinum and pemetrexed-based chemotherapy, showed diminished efficacy in second-generation ALK TKI refractory patients, with 29.7% ORR and 4.3 months mPFS [23]. Moreover, the effectiveness of immunotherapy in ALK+ NSCLC was more awful. Not only in the advanced setting [24], but the early-stage scenario [25], single-agent ICIs play a dispensable role in the treatment of ALK+ NSCLC. In addition, the employment of combined ICIs and chemotherapy did not change the prognosis as well: in the IMpower130 trial, atezolizumab combined with chemotherapy did not improve the 32 patients’ survival with EGFR mutated or ALK-rearranged NSCLC [26]. However, the case report showed impressive ICI efficacy in ALK+ individuals [27], suggesting some beneficial population might exist. Thus, it is of great interest to study the immunological characteristics of ALK-rearranged NSCLC.

TILs, especially CD8+ T cells, were thought to be an essential factor driving the poor response of ICIs in ALK+ NSCLC. Previous studies have shown that ALK+ NSCLC harbored a significantly lower CD8+ T cell infiltration than KRAS- or non-ALK/EGFR-mutated NSCLC [4,5], or a lower proportion of CD8+ T cells than EGFR-mutated NSCLC [9]. EML4-ALK transgenic mice models also showed a TIME with few infiltrating T cells, especially CD8+ T cells, in treatment-naïve tumors [28]. Consistent with the findings above, our bioinformatic analysis demonstrated the cold TIME of ALK+ tumors, characterized by less immune cells infiltration, higher proportions of naïve lymphocytes, and lower expression of immune-related genes. However, TLS was insufficiently studied in ALK+ tumors as a more organized peripheral lymphoid structure. For the first time, we focused on TLS in ALK+ tumors and evaluated immune cells in TLS and the tumor stroma independently. In line with our hypothesis, TLS and its cellular components showed more differential distribution than TILs and other stromal immune cells: only the densities of tB cells, tTh cells, and tCTL rather than their counterparts in the stroma, which we were attracted previously, decreased in ALK+ tumors significantly.

TLS-related variables also exhibited more substantial power on tumor progression than ETLS-ICs in our regression analysis. In the first equation, TLS density was identified as an independent factor of node metastasis and pStage in ALK+ tumors. This is consistent with the previous finding in NSCLC that TLS density was negatively associated with tumor stage, and the score based on TLS count was an independent favorable prognostic factor [29]. However, when we looked into the cellular components of TLS, tCTL%, and tB-cell density replaced TLS density to be the independently significant factors on node metastasis or pStage, respectively, which indicates that the cellular components of TLS, rather than the amounts of TLS themselves, more pronouncedly affected antitumor response. Previous studies have demonstrated that B cells in TLS were closely related with the protective immunity in patients with NSCLC, and the activated somatic hypermutation and class switch recombination machinery in the germinal centers of TLS directly led to the generation of plasma cells which are the final executors of humoral immunity [12]. Our results regarding the role of tB cells in pStage also emphasized the importance of TLS-based humoral immunity in ALK+ tumors, which showed predominance rather than cellular immunity, and this phenomenon might partly explain the poor performance of ICIs in ALK+ tumors, as blocking the PD-L1/PD-1 pathway does not help improve the humoral immunity efficiently. This hypothesis was supported by the eccentric and powerless increase of activated NK cells found in the bioinformatic analysis of ALK+ tumors.

The crosstalk among TIME features showed that the densities of TLS and main TLS-ICs were all positively correlated with eTh-cell density and the latter associated with other ETLS-ICs furtherly. The positive correlations between TLS and TILs have been reported in breast and colorectal cancer [30,31,32], and the possible mechanism was complicated. CXCL13 seemed to be a critical factor in this process. This chemokine was secreted by various lymphoid tissue-organizer cells, such as T helper cells or some stromal/immune cells, promoting the recruitment or expansion of lymphocytes, which secreted more chemokines, including IFN-γ, TNF-α, and et al., augmenting subsequent immune response [33]. Our result revealed that the link between TLS and TIL also existed in the relatively cold TIME of ALK+ tumors, but their causality could not be determined here. In the future, the composition of eTh-cell subgroups and their function should be evaluated, which might indicate some possible targets, making the TIME of ALK+ tumors hotter.

It is reported that tumor PD-L1 expression was also involved in the poor response to ICI-based therapy in ALK+ tumors, but the results of previous studies were not consistent. Either decreased or increased tumor PD-L1 expression could be seen in different ALK+ LUAD cohorts or animal models [4,7,9,34]. The variable PD-L1 expression level across studies may result from the different antibody clones applied, different quantitative methods, scenarios of specimens, or the relatively small sample size in every single research. In our study, PD-L1 expression was comparable between tumors with or without ALK rearrangement but correlated with the spatial organization of TLS within ALK+ LUAD: more tumors with intratumoral TLS expressed a significantly higher level of PD-L1. It was demonstrated that the function of TLS was site-specific in hepatocellular carcinoma and breast cancer [35], of which intratumoral TLS was related with a better outcome than the peritumoral TLS did. Although the process of humoral immunity could be detected in peritumoral TLS, due to the exclusion from tumor nests, they could not perform their roles as anti-tumor immune cells but as inductors of myeloid inflammatory response and promoted tumor progression [36]. Combined with the fact that PD-L1 expression would be induced by activated immune cells and inflammatory chemokines [37,38], we speculated that, similar to hepatocellular carcinoma and breast cancer above, the intratumoral TLS of ALK+ tumors were more activated than peritumoral TLS. Its more profound functions induced higher PD-L1 expression through the immunity-tumor interaction. And this phenomenon also indicated that ALK+ tumors with intratumoral TLS might be the entity that will benefit more from immunotherapy.

There were several limitations of the current study. First, we mainly focused on the quantity and distribution of TLS and ETLS-ICs, leaving the question open if varied functions related to different immune features. For further investigation, ALK+ tumor models with differential TLS formations would help explore the direct roles of TLS and the underlying mechanisms. Second, all patients in our study were early-stage LUAD. This is because accurate and comprehensive assessment of TLS and ETLS-ICs by IHC requires surgical specimens. It is noteworthy to mention that based on the poor response of ICIs in both early and advanced stage ALK+ LUAD, we believe that early and advanced ALK+ LUAD share some common immune characteristics accounting for the diminished efficacy of ICIs. New techniques are needed to evaluate TLS and other TIME elements in vivo, which makes evaluating the direct correlation between TIME and ICI response in advanced patients possible. Third, regarding the low incidence of ALK+ LUAD and the extensive work on the annotation and quantitative analysis of TLS one by one, the sample size of our study was relatively small. More cases and artificial intelligence approaches are expected to facilitate the profiling of TIME more comprehensively and efficiently in the future.

## 5. Conclusions

To date, this is the first study to assess the TIME of ALK+ LUAD, with particular emphasis on the features of TLS. Compared to ALK− LUAD, ALK+ LUAD possesses a relative “cold” TIME, with less TLS formation and TLS-ICs, which was closely correlated with tumor progression. Our results offer significant new insights for developing novel immunotherapy strategies in ALK+ NSCLC.

## Figures and Tables

**Table 1:** The cohort baseline before PSM.

Factor	ALK+	ALK−	*p*
Sex			
Male	17 (43.6)	6 (54.5)	0.733
Female	22 (56.4)	5 (45.5)	
Age at diagnosis			
<60 ys	22 (56.4)	8 (72.7)	0.489
≥60 ys	17 (43.6)	3 (27.3)	
Tumor size, cm			
Median (range)	1.5 (0.6, 5.0)	1.5 (0.5, 8.5)	0.474
Histologic differentiation			
Well	5 (12.8)	0 (0)	0.539
Moderate	22 (56.4)	8 (72.7)	
Poor	12 (30.8)	3 (27.3)	
Aerogenic spread, *n* (%)			
Negative	30 (76.9)	7 (63.6)	0.445
Positive	9 (23.1)	4 (36.4)	
LVI, *n* (%)			
Negative	29 (74.4)	8 (72.7)	1.000
Positive	10 (25.6)	3 (27.3)	
Pleural invasion, *n* (%)			
Negative	35 (89.7)	10 (90.9)	1.000
Positive	4 (10.3)	1 (9.1)	
Node metastasis, *n* (%)			
Negative	31 (79.5)	7 (63.6)	0.424
Positive	8 (20.5)	4 (36.4)	
pStage			
I	29 (74.4)	6 (54.5)	0.204
II	3 (7.7)	3 (27.3)	
III	7 (17.9)	2 (18.2)	

**Table 2:** The significance of immune parameters.

Feature	Significant Variables from Screening Models	Univariate Analysis				Multivariate Analysis			
		*p*	HR	95% CI		*p*	HR	95% CI	
				Lower	Upper			Lower	Upper
Node metastasis	TLS density	0.590	0.803	0.362	1.783	-			
	tCTL cell%	0.222	0.683	0.370	1.259	0.007	0.599	0.414	0.868
	eTh-cell density	0.895	1.060	0.448	2.507	-			
pStage	TLS density	0.762	1.229	0.323	4.685	-			
	tB-cell density	0.219	0.414	0.101	1.691	0.016	0.641	0.446	0.922
	eTh-cell density	0.577	1.241	0.581	2.654	-			

## Data Availability

Not applicable.

## Supplementary Materials

The following supporting information can be downloaded at: https://www.mdpi.com/article/10.3390/jcm11195935/s1, Table S1: Comparison of variables before and after PSM, n (%); Table S2: TIME comparison based on surgical samples; Table S3: The general characteristics of TLS in ALK+ LUAD, n (%); Table S4: The characteristics of TLS in ALK+ LUAD, median (range); Table S5: Densities of main TLS-ICs in ALK+ LUAD, median (range); Table S6: Proportions of main TLS-ICs in ALK+ LUAD, mean ± SEM; Table S7: Proportions of tTreg and tDC cells in ALK+ LUAD, n (%); Table S8: Proportions of tTAM in ALK+ LUAD, n (%); Table S9: Densities of stromal immune cells in ALK+ LUAD, median (range); Table S10: The significance of TLS and ETLS-ICs on tumor size in ALK+ LUAD; Table S11: The significance of TLS and ETLS-ICs on node metastasis in ALK+ LUAD; Table S12: The significance of TLS and ETLS-ICs on pStage in ALK+ LUAD; Table S13: PD-L1 expression and TIME in ALK+ LUAD, n (%); Figure S1: PSM between the GR and non-GR group.
