# Supplementary material for: Decreased Tertiary Lymphoid Structures in Lung Adenocarcinomas with ALK Rearrangements
Source: J Clin Med. 2022 Oct 8;11(19):5935. doi: 10.3390/jcm11195935 (PMC9572246; doi:10.3390/jcm11195935)
Supplement: Supplementary file 1 [file jcm-11-05935-s001.zip › jcm-1871134-supplementary.pdf]

Table S1. Comparison of variables before and after PSM, n (%)

Characteristic	Before			After		
	ALK+ (n=39)	ALK- (n=11)	P	ALK+ (n=9)	ALK- (n=9)	P
Sex						
Male	17 (43.6)	6 (54.5)	0.733	4 (44.4)	4 (44.4)	1.000
Female	22 (56.4)	5 (45.5)		5 (55.6)	5 (55.6)	
Age						
<60 ys	22 (56.4)	8 (72.7)	0.489	7 (77.8)	7 (77.8)	1.000
≥60 ys	17 (43.6)	3 (27.3)		2 (22.2)	2 (22.2)	
pStage						
I	29 (74.4)	6 (54.5)	0.204	6 (66.7)	6 (66.7)	1.000
II	3 (7.7)	3 (27.3)		2 (22.2)	2 (22.2)	
III	7 (17.9)	2 (18.2)		1 (11.1)	1 (11.1)	

Table S2. TIME comparison based on surgical samples

Factor		ALK+	ALK-	P
TLS				
	Absence	3 (33.3)	0 (0)	0.206
	Presence	6 (66.7)	9 (100.0)	
TLS Location				
	Peritumoral	3 (50.0)	4 (44.4)	1.000
	Intratumoral	3 (50.0)	5 (55.6)	
TLS density (/mm^2)		0.10 (0.04, 0.27)	0.34 (0.00, 1.98)	**0.026**
TLS/tumor *10^2		0.35 (0.17, 0.59)	1.61 (0.01, 6.92)	**0.012**
Single-TLS size *10^2 (mm^2)		3.02 (1.97, 4.70)	3.94 (2.10, 9.48)	0.272
tTh cell density (/mm^2)		57.65 (7.70, 141.55)	274.82 (0.87, 884.05)	**0.026**
tCTL density (/mm^2)		22.46 (5.16, 74.46)	172.83 (0.69, 464.59)	**0.018**
tB cell density (/mm^2)		36.08 (17.31, 101.84)	207.29 (1.17, 668.03)	**0.012**
tTh cell%		46.21±4.83	39.12±3.21	0.224
tCTL%		16.28±2.77	20.41±2.32	0.277
tB cell%		37.50±5.37	40.47±3.04	0.612
tTreg cell%				
	<1%	4 (66.7)	2 (22.2)	0.349
	1-10%	1 (16.7)	4 (44.4)	
	10-50%	1 (16.7)	3 (33.3)	
tDC%				
	<1%	1 (16.7)	1 (11.5)	1.000
	1-10%	4 (66.7)	5 (55.6)	
	10-50%	1 (16.7)	3 (33.3)	
tTAM%				
	<1%	0 (0)	0 (0)	0.400
	1-10%	5 (83.3)	9 (100.0)	
	10-50%	1 (16.7)	0 (0)	
eTh cell density *10^-2 (/mm^2)		32.60 (11.04, 65.21)	30.10 (12.04, 110.35)	0.730
eCTL density *10^-2 (/mm^2)		9.59 (4.48, 19.82)	12.15 (7.67, 35.80)	0.340
eTAM density *10^-2 (/mm^2)		5.12 (1.02, 35.86)	3.07 (2.05, 20.49)	0.340
PD-L1 (TC)				
	<1%	4 (44.4)	5 (55.6)	0.637
	≥1%, <10%	5 (55.6)	3 (33.3)	
	≥10%	0 (0)	1 (11.1)	

Table S3. The general characteristics of TLS in ALK+ LUAD, n (%)

Characteristic		Presence		P	Location		P
		+	-		Peritumoral	Intratumoral	
Sex							
	Male	13 (43.3)	4 (44.4)	1.000	4 (30.8)	9 (52.9)	0.283
	Female	17 (56.7)	5 (55.6)		9 (69.2)	8 (47.1)	
Age							
	<60 ys	19 (63.3)	3 (33.3)	0.142	8 (61.5)	7 (41.2)	0.462
	≥60 ys	11 (36.7)	6 (66.7)		5 (38.5)	10 (58.8)	
Tumor size							
	≤1.5cm	15 (50.0)	7 (77.8)	0.251	8 (61.5)	9 (52.9)	0.721
	>1.5cm	15 (50.0)	2 (22.2)		5 (38.5)	8 (47.1)	
Histologic differentiation							
	Well	3 (10.0)	2 (22.2)	0.662	1 (7.7)	2 (11.8)	0.087
	Moderate	17 (56.7)	5 (55.6)		5 (38.5)	12 (70.6)	
	Poor	10 (33.3)	2 (22.2)		7 (53.8)	3 (17.6)	
Aerogenic spread							
	Negative	24 (80.0)	6 (66.7)	0.406	10 (76.9)	14 (82.4)	1.000
	Positive	6 (20.0)	3 (33.3)		3 (23.1)	3 (17.6)	
LVI							
	Negative	22 (73.3)	7 (77.8)	1.000	8 (61.5)	14 (82.4)	0.242
	Positive	8 (26.7)	2 (22.2)		5 (38.5)	3 (17.6)	
Pleural invasion							
	Negative	26 (86.7)	9 (100.0)	0.556	10 (76.9)	16 (94.1)	0.290
	Positive	4 (13.3)	0 (0)		3 (23.1)	1 (5.9)	
Node metastasis							
	Negative	23 (76.7)	8 (88.9)	0.653	9 (69.2)	14 (82.4)	0.666
	Positive	7 (23.3)	1 (11.1)		4 (30.8)	3 (17.6)	
pStage							
	I	21 (70.0)	8 (88.9)	0.400	8 (61.5)	13 (76.5)	0.443
	II+III	9 (30.0)	1 (11.1)		5 (38.5)	4 (23.5)	

Table S4. The characteristics of TLS in ALK+ LUAD, median (range)

Characteristic		Density /mm^2	P	TLS/tumor *10^2	P	Single-TLS size *10^2 mm^2	P
Sex							
	Male	0.31 (0.05, 1.18)	0.509	1.30 (0.15, 8.45)	0.245	4.09 (2.70, 7.18)	**0.017**
	Female	0.25 (0.04, 1.80)		0.53 (0.17, 12.59)		2.64 (1.53, 26.40)	
Age							
	<60 ys	0.19 (0.04, 0.82)	0.420	0.50 (0.15, 4.33)	0.077	3.11 (1.53, 16.62)	0.057
	≥60 ys	0.26 (0.06, 1.80)		1.00 (0.19, 12.59)		4.70 (2.45, 26.40)	
Tumor size							
	≤1.5cm	0.27 (0.05, 1.80)	**0.006**	0.69 (0.15, 11.50)	0.089	2.77 (1.53, 7.18)	0.202
	>1.5cm	0.09 (0.04, 0.51)		0.32 (0.17, 12.59)		3.96 (1.89, 26.40)	
Histologic differentiation							
	Well	0.25 (0.05, 0.31)	0.532	0.69 (0.15, 1.46)	0.532	2.77 (2.70, 4.70)	0.292
	Moderate	0.27 (0.04, 1.80)		0.53 (0.17, 12.59)		3.11 (1.53, 26.40)	
	Poor	0.16 (0.04, 1.18)		0.60 (0.20, 8.45)		4.44 (2.16, 16.62)	
Aerogenic spread							
	Negative	0.22 (0.04, 1.80)	0.116	0.51 (0.15, 12.59)	0.050	3.00 (1.53, 26.40)	0.104
	Positive	0.41 (0.08, 0.82)		1.88 (0.29, 4.33)		4.43 (3.63, 6.81)	
LVI							
	Negative	0.26 (0.04, 1.80)	0.945	0.56 (0.15, 12.59)	0.534	3.26 (1.53, 26.40)	0.298
	Positive	0.20 (0.04, 0.82)		0.60 (0.20, 4.33)		3.93 (2.16, 16.62)	
Pleural invasion							
	Negative	0.27 (0.04, 1.80)	0.746	0.59 (0.15, 12.59)	0.576	3.57 (1.53, 26.40)	0.617
	Positive	0.16 (0.09, 0.27)		0.54 (0.18, 1.00)		3.06 (2.05, 4.90)	
Node metastasis							
	Negative	0.27 (0.04, 1.80)	0.666	0.53 (0.15, 12.59)	0.848	3.63 (1.53, 26.40)	0.737
	Positive	0.19 (0.04, 0.82)		0.60 (0.20, 4.33)		3.50 (2.45, 16.62)	
pStage							
	I	0.27 (0.04, 1.80)	0.349	0.59 (0.15, 12.59)	0.790	3.63 (1.53, 26.40)	0.756
	II+III	0.12 (0.04, 0.82)		0.58 (0.20, 4.33)		3.50 (2.45, 16.62)	

Table S5. Densities of main TLS-ICs in ALK+ LUAD, median (range)

Characteristic		tTh cell /mm^2	P	tCTL cell /mm^2	P	tB cell /mm^2	P
Sex							
	Male	76.48 (9.31, 525.91)	0.773	39.08 (6.01, 413.35)	0.536	139.52 (20.81, 1259.50)	0.320
	Female	68.96 (5.35, 1914.30)		30.13 (5.16, 792.93)		65.39 (7.34, 1688.96)	
Age							
	<60 ys	62.11 (5.35, 426.70)	0.158	30.13 (5.16, 210.35)	0.268	40.52 (7.34, 823.40)	0.085
	≥60 ys	114.45 (9.31, 1914.30)		44.40 (6.01, 792.93)		139.52 (20.81, 1688.96)	
Tumor size							
	≤1.5cm	114.45 (19.75, 1914.30)	**0.007**	50.64 (9.55, 792.93)	0.056	139.52 (28.72, 1688.96)	**0.004**
	>1.5cm	44.00 (5.35, 1065.41)		27.52 (5.16, 622.65)		33.49 (7.34, 1487.39)	
Histologic differentiation							
	Well	106.18 (19.75, 252.37)	0.844	17.48 (9.55, 25.63)	0.323	139.52 (28.72, 150.44)	0.949
	Moderate	76.48 (7.70, 1914.30)		39.08 (5.16, 792.93)		74.08 (7.34, 1688.96)	
	Poor	60.57 (5.35, 525.91)		28.83 (9.90, 413.35)		46.71 (14.39, 1259.50)	
Aerogenic spread							
	Negative	62.61 (5.35, 1914.30)	0.432	27.76 (5.16, 792.93)	0.065	56.99 (7.34, 1688.96)	0.082
	Positive	95.47 (43.97, 426.70)		89.80 (30.13, 210.35)		204.90 (39.05, 823.40)	
LVI							
	Negative	69.80 (5.76, 1914.30)	0.982	33.53 (5.16, 792.93)	0.836	69.73 (7.34, 1688.96)	0.836
	Positive	91.18 (5.35, 426.70)		28.83 (13.48, 210.35)		71.18 (19.72, 823.40)	
Pleural invasion							
	Negative	85.47 (5.35, 1914.30)	0.271	33.53 (5.16, 792.93)	0.298	87.16 (14.39, 1688.96)	0.177
	Positive	43.17 (24.95, 113.41)		22.46 (9.54, 44.40)		38.24 (7.34, 101.84)	
Node metastasis							
	Negative	68.96 (7.70, 1914.30)	0.811	31.00 (5.16, 792.93)	0.848	74.08 (7.34, 1688.96)	0.598
	Positive	76.48 (5.35, 426.35)		30.73 (9.90, 210.35)		40.52 (14.39, 823.40)	
pStage							
	I	94.47 (7.70, 1914.30)	0.283	39.08 (5.16, 792.93)	0.372	100.24 (7.34, 1688.96)	0.193
	II+III	52.19 (5.35, 426.70)		27.52 (6.84, 210.35)		33.49 (14.39, 823.40)	

Table S6. Proportions of main TLS-ICs in ALK+ LUAD, mean±SEM

Characteristic		tTh cell%	P	tCTL%	P	tB cell%	P
Sex							
	Male	32.09±2.52	**0.025**	16.20±1.71	0.781	51.71±3.26	**0.022**
	Female	44.52±4.15		15.56±1.53		39.93±3.42	
Age							
	<60 ys	40.09±4.03	0.661	16.26±1.35	0.631	43.66±3.77	0.495
	≥60 ys	37.48±3.32		15.11±2.06		47.41±2.87	
Tumor size							
	≤1.5cm	39.75±2.65	0.830	13.98±1.41	0.100	46.27±2.27	0.643
	>1.5cm	38.51±5.04		17.69±1.65		43.80±4.75	
Histologic differentiation							
	Well	44.88±7.75	0.517	8.00±2.89	**0.030**	47.12±5.28	0.835
	Moderate	40.65±3.53		15.72±1.08		43.63±3.53	
	Poor	34.82±5.52		18.39±2.30		46.79±4.96	
Aerogenic spread							
	Negative	41.18±3.20	0.146	15.38±1.24	0.422	43.45±2.88	0.227
	Positive	30.94±4.63		17.68±2.69		51.38±5.65	
LVI							
	Negative	39.92±3.05	0.650	15.28±1.25	0.424	44.80±2.90	0.885
	Positive	36.97±6.64		17.35±2.47		45.67±5.93	
Pleural invasion							
	Negative	37.73±3.04	0.208	15.37±1.11	0.299	46.90±2.70	0.066
	Positive	48.22±6.01		18.86±4.60		32.92±6.04	
Node metastasis							
	Negative	39.38±2.45	0.873	15.88±1.21	0.944	44.73±2.53	0.839
	Positive	38.30±9.47		15.69±2.89		46.01±7.87	
pStage							
	I	39.79±2.52	0.727	15.62±1.25	0.769	44.60±2.45	0.803
	II+III	37.60±7.57		16.35±2.45		46.05±6.79	

Table S7. Proportions of tTreg and tDC cells in ALK+ LUAD, n (%)

Characteristic		tTreg cell		P	tDC			P
	<1%	1%-10%	10%-50%		<1%	1%-10%	10%-50%	
Sex								
Male	6 (33.3)	5 (55.6)	2 (66.7)	0.467	1 (33.3)	9 (47.4)	3 (37.5)	0.867
Female	12 (66.7)	4 (44.4)	1 (33.3)		2 (66.7)	10 (52.6)	5 (62.5)	
Age								
<60 ys	12 (66.7)	5 (55.6)	2 (66.7)	0.853	2 (66.7)	11 (57.9)	6 (75.0)	0.845
≥60 ys	6 (33.3)	4 (44.4)	1 (33.3)		1 (33.3)	8 (42.1)	2 (25.0)	
Tumor size								
≤1.5cm	10 (55.6)	4 (44.4)	1 (33.3)	0.763	2 (66.7)	10 (52.6)	3 (37.5)	0.750
>1.5cm	8 (44.4)	5 (55.6)	2 (66.7)		1 (33.3)	9 (47.4)	5 (62.5)	
Histologic differentiation								
Well	1 (5.6)	1 (11.1)	1 (33.3)	0.406	1 (33.3)	1 (5.3)	1 (12.5)	0.264
Moderate	12 (66.7)	4 (44.4)	1 (33.3)		2 (66.7)	12 (63.2)	3 (37.5)	
Poor	5 (27.8)	4 (44.4)	1 (33.3)		0 (0)	6 (31.6)	4 (50.0)	
Aerogenic spread								
Negative	16 (88.9)	5 (55.6)	3 (100.0)	0.136	3 (100.0)	15 (78.9)	6 (75.0)	1.000
Positive	2 (11.1)	4 (44.4)	0 (0)		0 (0)	4 (21.1)	2 (25.0)	
LVI								
Negative	15 (83.3)	5 (55.6)	2 (66.7)	0.265	3 (100.0)	14 (73.7)	5 (62.5)	0.703
Positive	3 (16.7)	4 (44.4)	1 (33.3)		0 (0)	5 (26.3)	3 (37.5)	
Pleural invasion								
Negative	16 (88.9)	8 (88.9)	2 (66.7)	0.531	3 (100.0)	18 (94.7)	5 (62.5)	0.087
Positive	2 (11.1)	1 (11.1)	1 (33.3)		0 (0)	1 (5.3)	3 (37.5)	
Node metastasis								
Negative	14 (77.8)	7 (77.8)	2 (66.7)	1.000	3 (100.0)	15 (78.9)	5 (62.5)	0.543
Positive	4 (22.2)	2 (22.2)	1 (33.3)		0 (0)	4 (21.1)	3 (37.5)	
pStage								
I	13 (72.2)	7 (77.8)	1 (33.3)	0.364	3 (100.0)	13 (68.4)	5 (62.5)	0.704
II+III	5 (27.8)	2 (22.2)	2 (66.7)		0 (0)	6 (31.6)	3 (37.5)	

Table S8. Proportions of tTAM in ALK+ LUAD, n (%)

Characteristic		tTAM			P
		<1%	1%-10%	10%-50%	
Sex					
	Male	3 (75.0)	9 (36.0)	1 (100.0)	0.181
	Female	1 (25.0)	16 (64.0)	0 (0)	
Age					
	<60 ys	3 (75.0)	16 (64.0)	0 (0)	0.536
	≥60 ys	1 (25.0)	9 (36.0)	1 (100.0)	
Tumor size					
	≤1.5cm	3 (75.0)	12 (48.0)	0 (0)	0.598
	>1.5cm	1 (25.0)	13 (52.0)	1 (100.0)	
Histologic differentiation					
	Well	1 (25.0)	2 (8.0)	0 (0)	0.738
	Moderate	2 (50.0)	14 (56.0)	1 (100.0)	
	Poor	1 (25.0)	9 (36.0)	0 (0)	
Aerogenic spread					
	Negative	2 (50.0)	21 (84.0)	1 (100.0)	0.344
	Positive	2 (50.0)	4 (16.0)	0 (0)	
LVI					
	Negative	2 (50.0)	19 (76.0)	1 (100.0)	0.487
	Positive	2 (50.0)	6 (24.0)	0 (0)	
Pleural invasion					
	Negative	4 (100.0)	21 (84.0)	1 (100.0)	1.000
	Positive	0 (0)	4 (16.0)	0 (0)	
Node metastasis					
	Negative	4 (100.0)	18 (72.0)	1 (100.0)	0.652
	Positive	0 (0)	7 (28.0)	0 (0)	
pStage					
	I	4 (100.0)	16 (64.0)	1 (100.0)	0.496
	II+III	0 (0)	9 (36.0)	0 (0)	

Table S9. Densities of stromal immune cells in ALK+ LUAD, median (range)

Characteristic		eTh cell *10^-2 /mm^2	P	eCTL cell *10^-2 /mm^2	P	eTAM *10^-2 /mm^2	P
TLS							
	Absence	34.11 (11.04, 62.70)	0.635	5.11 (1.92, 19.18)	**0.046**	4.10 (2.05, 10.25)	0.366
	Presence	29.60 (10.53, 95.31)		10.23 (3.20, 20.46)		3.67 (1.02, 35.86)	
Sex							
	Male	27.59 (10.53, 79.25)	0.377	8.31 (4.48, 20.46)	0.585	4.10 (1.02, 35.86)	0.154
	Female	35.87 (11.04, 95.31)		9.91 (1.92, 18.54)		3.07 (1.02, 10.25)	
Age							
	<60 ys	33.36 (11.04, 79.25)	0.878	10.87 (3.84, 20.46)	0.124	3.93 (1.02, 35.86)	0.967
	≥60 ys	27.59 (10.53, 95.31)		7.67 (1.92, 19.18)		4.10 (1.02, 10.25)	
Tumor size							
	≤1.5cm	32.60 (11.04, 79.25)	0.475	7.99 (1.92, 19.18)	0.077	3.07 (1.02, 8.20)	**0.014**
	>1.5cm	27.59 (10.53, 95.31)		11.50 (5.11, 20.46)		4.10 (1.02, 35.86)	
Histologic differentiation							
	Well	32.60 (13.04, 39.13)	0.093	4.48 (3.20, 13.43)	0.088	3.07 (2.05, 6.15)	0.348
	Moderate	40.13 (10.53, 95.31)		10.23 (1.92, 19.18)		3.33 (1.02, 10.25)	
	Poor	18.31 (11.04, 65.21)		7.99 (4.48, 20.46)		4.10 (2.05, 35.86)	
Aerogenic spread							
	Negative	33.36 (10.53, 95.31)	0.255	8.63 (1.92, 19.82)	0.588	3.67 (1.02, 35.86)	0.706
	Positive	23.07 (11.04, 57.69)		10.23 (5.11, 20.46)		4.10 (2.05, 10.25)	
LVI							
	Negative	34.11 (10.53, 95.31)	0.174	7.67 (1.92, 19.18)	0.365	3.59 (1.02, 10.25)	0.245
	Positive	20.82 (11.04, 65.21)		10.23 (4.48, 20.46)		4.10 (2.05, 35.86)	
Pleural invasion							
	Negative	32.60 (10.53, 95.31)	0.381	10.22 (1.92, 20.46)	0.841	4.10 (1.02, 10.25)	0.704
	Positive	16.55 (12.54, 65.21)		7.67 (4.48, 19.82)		3.84 (2.05, 35.86)	
Node metastasis							
	Negative	32.60 (10.53, 95.31)	0.235	9.59 (1.92, 20.46)	0.746	4.10 (1.02, 35.86)	0.932
	Positive	22.57 (11.04, 52.67)		8.95 (5.11, 18.54)		3.93 (2.05, 10.25)	
pStage							
	I	32.60 (11.04, 95.31)	0.232	9.59 (1.92, 20.46)	0.646	4.10 (1.02, 10.25)	0.887
	II+III	22.57 (10.53, 65.21)		8.95 (5.11, 19.82)		3.93 (1.02, 35.86)	

Table S10. The significance of TLS and ETLS-ICs on tumor size in ALK+ LUAD

Model	Variable		Univariate analysis				Multivariate analysis			
			P	HR	95% CI		P	HR	95% CI	
					Lower	Upper			Lower	Upper
Model 1	TLS density		0.214	0.341	0.062	1.861	-			
	TLS/tumor		1.000	1.000	0.146	6.839				
	Single-TLS size		0.107	1.942	0.866	4.355				
	TLS location	PT	Ref	1.000	-	-				
		IT	0.085	5.117	0.796	32.878				
Model 2	tB cell%		0.391	1.371	0.666	2.824	-			
	tTh cell%		0.827	1.085	0.522	2.254				
	tCTL%		0.207	1.704	0.745	3.896				
	tB-cell density		0.345	0.394	0.057	2.726				
	tTh-cell density		0.721	0.758	0.166	3.466				
	tCTL density		0.869	1.191	0.148	9.607				
Model 3	eTh-cell density		**0.023**	0.480	0.255	0.904	-			
	eCTL density		0.367	1.360	0.697	2.654				
	eTAM density		0.172	1.469	0.846	2.549				

PT: peritumoral; IT: intratumoral.

Table S11. The significance of TLS and ETLS-ICs on node metastasis in ALK+ LUAD

Model	Variable		Univariate analysis				Multivariate analysis			
			P	HR	95% CI		P	HR	95% CI	
					Lower	Upper			Lower	Upper
Model 1	TLS density		0.540	0.597	0.115	3.100	**0.010**	0.621	0.432	0.894
	TLS/tumor		0.735	1.413	0.191	10.454				
	Single-TLS size		0.667	0.859	0.429	1.721				
	TLS location	PT	Ref	1.000	-	-				
		IT	0.364	0.469	0.091	2.410				
Model 2	tB cell%		0.354	1.450	0.661	3.182				
	tTh cell%		0.277	0.611	0.251	1.487				
	tCTL%		0.432	0.692	0.276	1.735	**0.007**	0.599	0.414	0.868
	tB-cell density		0.137	0.177	0.018	1.731				
	tTh-cell density		0.280	2.757	0.439	17.332				
	tCTL density		0.645	1.715	0.172	17.088				
Model 3	eTh-cell density		0.074	0.533	0.267	1.064	**0.001**	0.577	0.416	0.802
	eCTL density		0.577	1.246	0.575	2.698				
	eTAM density		0.675	0.875	0.469	1.631				

PT: peritumoral; IT: intratumoral.

Table S12. The significance of TLS and ETLS-ICs on pStage in ALK+ LUAD

Model	Variable		Univariate analysis				Multivariate analysis			
			P	HR	95% CI		P	HR	95% CI	
					Lower	Upper			Lower	Upper
Model 1	TLS density		0.557	0.631	0.136	2.93	**0.025**	0.683	0.489	0.953
	TLS/tumor		0.794	1.282	0.199	8.258				
	Single-TLS size		0.872	0.948	0.494	1.82				
	TLS location	PT	Ref	1.000	-	-				
		IT	0.497	0.589	0.128	2.711				
Model 2	tB cell%		0.132	1.843	0.832	4.085				
	tTh cell%		0.423	0.719	0.320	1.613				
	tCTL%		0.577	0.784	0.333	1.845				
	tB-cell density		**0.040**	0.078	0.007	0.885	**0.016**	0.641	0.446	0.922
	tTh-cell density		0.416	2.041	0.366	11.385				
	tCTL density		0.288	3.421	0.354	33.069				
Model 3	eTh-cell density		0.097	0.587	0.314	1.100	**0.004**	0.653	0.489	0.871
	eCTL density		0.522	1.262	0.620	2.568				
	eTAM density		0.695	0.891	0.501	1.586				

PT: peritumoral; IT: intratumoral.

Table S13. PD-L1 expression and TIME in ALK+ LUAD, n (%)

Factor		TC<1%	1%≤TC<10%	TC≥10%	P
TLS					
	Absence	4 (21.1)	3 (18.8)	2 (50.0)	0.413
	Presence	15 (78.9)	13 (81.3)	2 (50.0)	
Location					
	Peritumoral	10 (66.7)	3 (23.1)	0 (0)	**0.023**
	Intratumoral	5 (33.3)	10 (76.9)	2 (100.0)	
TLS density (/mm^2)		0.12 (0.04, 1.18)	0.27 (0.04, 1.80)	0.41 (0.37, 0.45)	0.113
TLS/tumor *10^2		0.49 (0.15, 8.45)	1.00 (0.17, 12.59)	2.12 (1.73, 2.52)	0.262
Single-TLS size *10^2 (mm^2)		2.89 (1.85, 16.62)	4.09 (1.53, 26.40)	5.34 (3.88, 6.81)	0.113
Density *10^2 (/mm^2)					
	tTh cell	56.14 (5.35, 525.91)	94.47 (7.70, 1914.30)	125.78 (114.45, 137.10)	0.410
	tCTL	27.52 (6.84, 413.35)	40.65 (5.16, 792.93)	145.01 (135.21, 154.81)	0.149
	tB cell	40.52 (14.39, 1259.50)	100.24 (7.34, 1688.96)	331.03 (218.98, 443.07)	0.168
Proportions in TLS					
	tB cell%	44.96±3.60	43.29±4.18	56.96±6.65	0.463
	tTh cell%	38.86±4.00	42.00±4.22	22.54±3.24	0.254
	tCTL%	16.19±1.78	14.71±1.50	20.50±3.40	0.457
tTreg cell%					
	<1%	8 (53.3)	9 (69.2)	1 (50.0)	0.561
	1-10%	6 (40.0)	2 (15.4)	1 (50.0)	
	10-50%	1 (6.7)	2 (15.4)	0 (0)	
tDC%					
	<1%	0 (0)	3 (23.1)	0 (0)	0.284
	1-10%	11 (73.3)	6 (46.2)	2 (100.0)	
	10-50%	4 (26.7)	4 (30.8)	0 (0)	
tTAM%					
	<1%	1 (6.7)	1 (7.7)	2 (100.0)	**0.029**
	1-10%	13 (86.7)	12 (92.3)	0 (0)	
	10-50%	1 (6.7)	0 (0)	0 (0)	
eTh-cell density *10^-2 (/mm^2)		26.59 (10.53, 65.21)	42.64 (12.54, 95.31)	35.87 (11.04, 60.19)	0.152
eCTL density *10^-2 (/mm^2)		8.31 (3.20, 19.82)	10.55 (1.92, 19.18)	7.67 (5.11, 20.46)	0.630
eTAM density *10^-2 (/mm^2)		4.10 (1.02, 35.86)	3.84 (1.02, 8.20)	3.07 (2.05, 10.25)	0.742

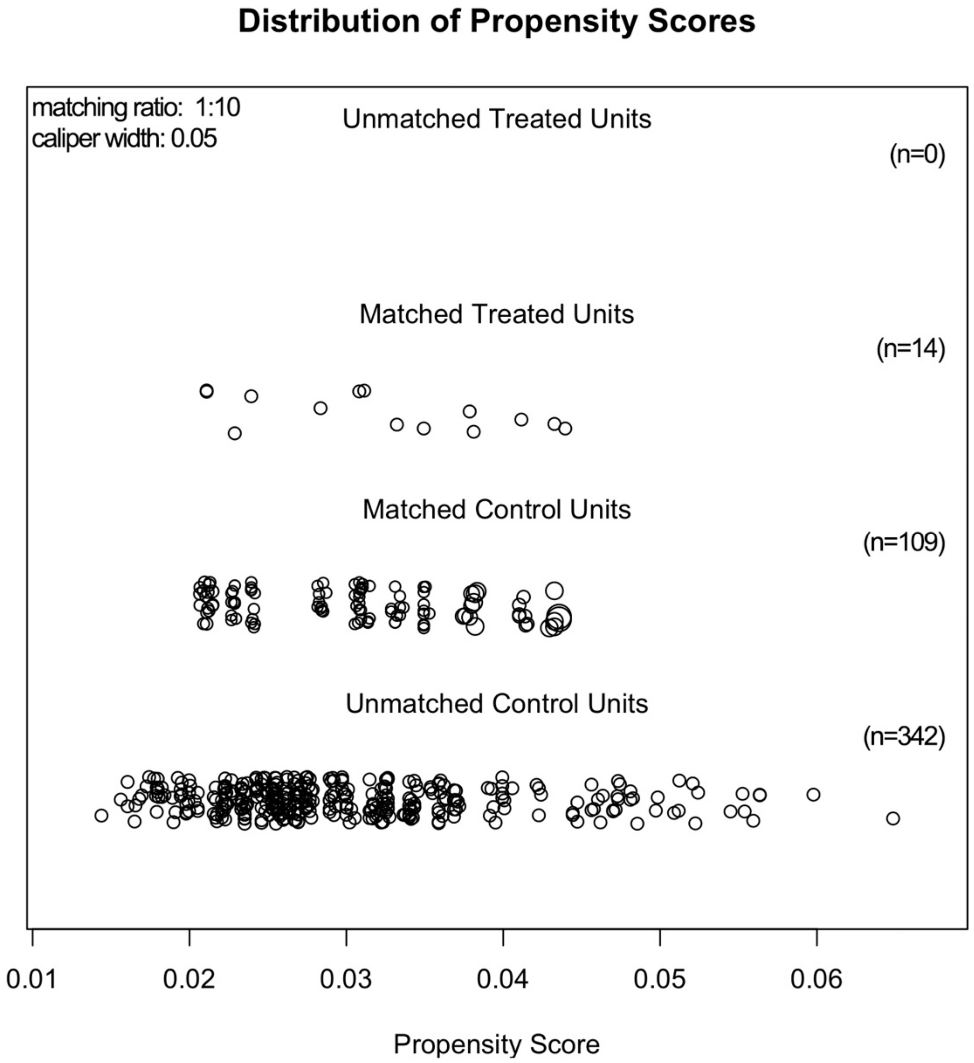

Figure S1. PSM between the GR and non-GR group
